# Spa therapy and peripheral serotonin and dopamine function: a systematic review

**DOI:** 10.1007/s00484-023-02579-0

**Published:** 2023-11-10

**Authors:** Isabel Gálvez, Antonella Fioravanti, Eduardo Ortega

**Affiliations:** 1 Immunophysiology Research Group, Instituto Universitario de Investigación Biosanitaria de Extremadura (INUBE), 06006 Badajoz, Spain; 2 Departamento de Enfermería, Facultad de Medicina y Ciencias de la Salud, Universidad de Extremadura, 06006 Badajoz, Spain; 3 Organisation Mondiale du Thermalisme (OMTh) - World Hydrothermal Organization, Sede Palazzo Terme, via Vittorio Emanuele, 38056 Levico Terme, Italy; 4 Departamento de Fisiología, Facultad de Ciencias, Universidad de Extremadura, 06071 Badajoz, Spain

**Keywords:** Serotonin, Dopamine, Spa therapy, Balneotherapy, Mud therapy, Endocrine response

## Abstract

Spa therapy consists of multiple techniques based on the healing effects of water, including hydrotherapy, balneotherapy, and mud therapy, often combined with therapeutic exercises, massage, or physical therapy. Balneotherapy is a clinically effective complementary approach in the treatment of low-grade inflammation- and stress-related pathologies, especially rheumatic conditions due to its anti-inflammatory properties. The main objective of this investigation was to conduct a systematic review analyzing the available evidence on the effect of spa therapy on serotonin and dopamine function. The databases PubMed, Web of Science, Scopus, and Cochrane Central Register of Controlled Trials (CENTRAL) were used from June to July 2023. Exclusion criteria were (1) articles not written in English, (2) full text not available, (3) article not related to the objective of the review. JADAD scale was used for methodological quality evaluation. Four studies were included in the systematic review. Two studies were related to serotonin in healthy individuals, one to serotonin in fibromyalgia, and one to dopamine in healthy individuals. One of the studies evaluated hydrotherapy, another one balneotherapy and mud-bath therapy, and the other two assessed balneotherapy interventions. Studies were very heterogeneous, and their methodological quality was low, making it difficult to draw clear conclusions regarding the effect of spa therapy on peripheral serotonin and dopamine function. The findings of this review highlight the lack of studies evaluating these neurotransmitters and hormones in the context of spa therapy. Further research is needed to evaluate the potential effects of these therapies on serotonin or dopamine function.

## Introduction

Spa therapy consists of multiple techniques based on the therapeutic application methods of water and peloids, including hydrotherapy and balneotherapy combined with rehabilitation, massage, or physical therapy (Bender et al. 2005; Gutenbrunner et al. 2010).

Balneotherapy, involving the use of natural mineral and/or thermal waters, gases, and peloids or muds, represents a clinically effective complementary approach in the treatment of several low-grade inflammation- and stress-related pathologies, especially rheumatic conditions, due to its anti-inflammatory, antioxidant, and chondroprotective properties (Fioravanti et al. 2013; Gálvez et al. 2018a; Cheleschi et al. 2022). A recently proposed mechanism of effectiveness of balneotherapy with peloids, or pelotherapy, in osteoarthritis (OA) patients is a neuroendocrine-immune stabilization that involves circulating inflammatory cytokines, cortisol, extracellular heat shock proteins (eHsp72), and regulatory T cell phenotype and neutrophil- and monocyte-mediated innate responses (Ortega et al. 2017; Gálvez et al. 2018b, 2020). Despite these recent advances, the effects of spa therapy and its different modalities on many physiologically relevant biomarkers, particularly those related to the neuroendocrine and immune response, have not yet been completely elucidated. Since the immune and nervous systems participate in a fine-tuned bidirectional crosstalk mediated by cytokines, neurotransmitters, and hormones, in the present work, we focus on the neurotransmitters/hormones serotonin and dopamine as key transmitters between the nervous and the immune system and thus potential mediators that may be involved in the anti-inflammatory effects of balneotherapy.

Serotonin (5-hydroxytryptamine, 5-HT) is a well-known neurotransmitter that mediates a diverse range of biological and behavioral processes such as circadian rhythms, food intake, sleep, reproductive activity, pain, cognition, mood, and anxiety/stress. Outside the central nervous system, peripheral serotonin acts as a hormone and also plays a fundamental, but lesser-known role, in many other physiological functions (bone mass, tissue regeneration—cell proliferation and wound healing—coagulation, angiogenesis, hematopoiesis gastrointestinal function, thermoregulation, and immunity) (Wu et al. 2019; Nieto et al. 2021). While the immune system was previously seen as unrelated to serotonin, recent findings have made it increasingly clear that peripheral serotonin plays a key part in the regulation of inflammation and immunity (Wu et al. 2019). In fact, the direct involvement of altered peripheral serotonin levels in the development and resolution of immunity/inflammation-related pathologies has been demonstrated (Nieto et al. 2020, 2021), although the exact role of peripheral serotonin in rheumatic diseases is yet to be elucidated.

Dopamine or 3-hydroxytyramine is a catecholamine neurotransmitter with well-recognized functions in the central nervous system, involving neurological processes such as motor control, emotion, behavior, cognition, learning, and reward (Matt and Gaskill 2020; Feng and Lu 2021). Moreover, dopamine is also present in peripheral tissues, regulating numerous functions in a variety of peripheral systems including gastrointestinal motility, hormone release, glucose homeostasis, body weight, blood pressure, sodium balance, adrenal and renal function, and immune function (Pinoli et al. 2017; Matt and Gaskill 2020). Dysfunction of the systemic or local dopaminergic system during inflammation has been found in various inflammatory and rheumatic diseases (Feng and Lu 2021); however, the precise mechanisms and functions of dopaminergic signaling, especially dopaminergic immunoregulation, have yet to be fully understood.

Bearing in mind the pleiotropic effects of spa therapy, affecting different systems of the body (musculoskeletal, integumentary, nervous, cardiovascular, respiratory, digestive, urinary, endocrine…) in which serotonin and dopamine are involved, and considering that the main mechanism of effectiveness of these therapies is through neuroendocrine and immune/inflammatory pathways, understanding the modulatory effects of spa therapy on serotonin and dopamine regulation becomes particularly relevant since it holds great potential for therapeutic interventions, especially in the context of rheumatic diseases and other pathologies characterized by altered serotonin and dopamine levels.

Taking all of this into account, the aim of this study was to review and critically examine the available evidence on the effect of spa therapy (and its modalities, including balneotherapy, pelotherapy, and hydrotherapy) on serotonin and dopamine function.

## Material and methods

A systematic review regarding the effect of balneotherapy on serotonin and dopamine concentrations was performed. The PRISMA Statement determines the set of items to include in systematic reviews and meta-analyses. We followed the PRISMA 2020 Statement and Checklist (Page et al. 2021). The review protocol was registered at PROSPERO International prospective register of systematic reviews under registration code CRD42023390530.

**Fig. 1:**
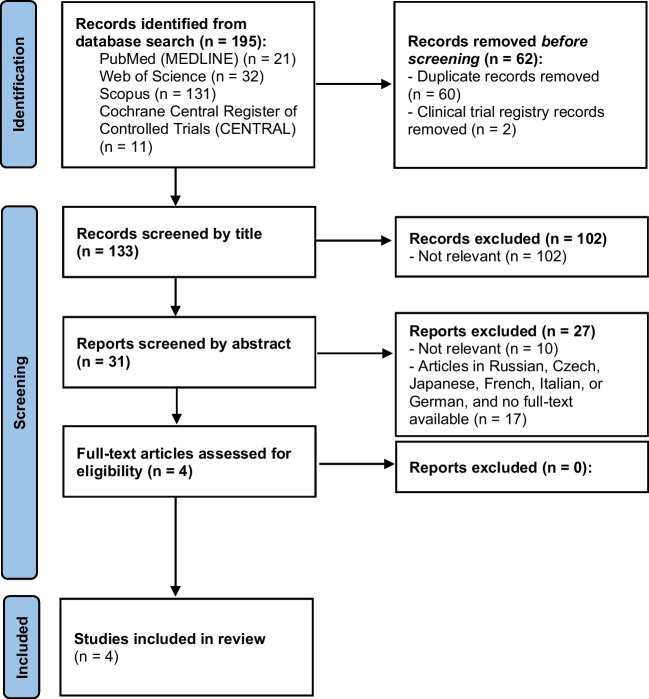
Flow diagram of the systematic review

From June to July 2023, the databases PubMed (MEDLINE), Web of Science, Scopus, and Cochrane Central Register of Controlled Trials (CENTRAL) were used to collect the articles. The search terms were as follows: (“serotonin” OR “5-HT”) AND (“balneotherapy” OR “crenotherapy” OR “pelotherapy” OR “mud therapy” OR “spa therapy” OR “hydrotherapy” OR “creno-balneotherapy”), as well as (“dopamine”) AND (“balneotherapy” OR “crenotherapy” OR “pelotherapy” OR “mud therapy” OR “spa therapy” OR “hydrotherapy” OR “creno-balneotherapy”). Due to the low number of studies retrieved, no filters or year limits were applied. Duplicated studies were manually excluded. The articles were included in the review according to the following inclusion criteria: (1) spa therapy and its modalities were applied to the participants in the study; (2) the study analyzed and reported variables related to serotonin or dopamine. Moreover, exclusion criteria were as follows: (1) articles not written in English, (2) full text not available, (3) article not related to the objective of the systematic review. The selection process is shown in Fig. 1, which shows the flow diagram of the systematic review conforming to the PRISMA 2020 statement. Two researchers independently screened the scientific literature, selected the studies, and extracted data.

The data extraction process was conducted following the PICOS approach. PICOS is the acronym for participants, intervention, comparison, results, and study design. Therefore, this information was collected from each study included in the review, as shown in the “Results” section. Regarding participants, group sample size, age, and pathology were included. Interventions were presented by describing the intervention type, duration, frequency, and characteristics of water and/or peloids (composition, temperature, place...). The outcome assessment included variables analyzed (related to serotonin and dopamine) and measurement methods used, comparison (baseline, control groups, etc.), and results.

Furthermore, the methodological quality of the studies was assessed using the JADAD scale (Jadad et al. 1996). It is a scale that is easy to use and contains many of the important elements that have been shown to correlate with bias; moreover, it has known reliability and external validity. It is a questionnaire comprised of three questions: (1) Was the study described as randomized? (2) Was the study described as double-blind? (3) Was there a description of withdrawals and dropouts? That can be answered with either a yes (1 point) or a no (0 points). 1 additional point is given if the method of randomization was described in the paper and that method was appropriate. Another additional point is given if the method of blinding was described, and it was appropriate. 1 point is deducted if the method of randomization was described but was inappropriate, and another point is deducted if the method of blinding was described but was inappropriate. Thus, the assessed study can obtain a total score between 0 (very poor quality) and 5 (rigorous), with scores lower than 3 indicating poor methodological quality of the study. Two researchers independently carried out the quality assessment.

## Results

As reported in Fig. 1, on the basis of our selection process, only four articles were included in the systematic review. Firstly, a total of 195 articles were identified (21 in PubMed, 32 in Web of Science, 131 in Scopus, and 11 in Cochrane Central Register of Controlled Trials) using the database search strategy described in the “Material and methods” section. Before screening, 21 duplicate articles were excluded, leaving 32 articles to be screened by title or abstract. Twenty-eight studies were excluded because they were not relevant to the objective of the systematic review (they did not comply with inclusion criteria) (*n* = 16), or they were not written in English, and moreover, full text was not available (*n* = 12). A full-text analysis of 4 articles was then conducted, and all of them accomplished the inclusion and exclusion criteria. Therefore, all four articles were included in the review: three of them were related to serotonin and the other one to dopamine.

Table 1 describes the main characteristics of the studies and their participants that were included in the systematic review. The review includes a sample size of 161 participants divided into the following groups: healthy group that underwent a control intervention (*n* = 58), healthy individuals experimental group (*n* = 62), and fibromyalgia patients experimental group (*n* = 41). Considering all groups, ages ranged from 22 to 69 years, and the mean age was 45.5 years old.

**Table 1:** Characteristics of the studies included in the systematic review and their participants

Study	Study design	Sample size	Pathology	Age
Kurabayashi et al. 2001	Interventional study	Total: *n* = 20 Intervention: *n* = 12 Control intervention: *n* = 8	Healthy volunteers	Intervention: 22–35 years range Control: -
Marazziti et al. 2007	Interventional study	Total: *n* = 40 Intervention: *n* = 20 Control intervention: *n* = 20	Healthy volunteers	Intervention: 27–54 years range, mean ± SD 44.5 ± 7.6 Control: 25–54 years range, mean ± SD: 42.4 ± 5.9
Baroni et al. 2012	Interventional study	Total: *n *= 60 Intervention: n = 30 Control intervention: *n* = 30	Healthy volunteers	Intervention: 24–50 years range, mean ± SD 39.4 ± 8 Control: 24-54 years range, mean ± SD: 40.2 ± 7.4
Bazzichi et al. 2013	Randomized controlled trial	Total: *n* = 41 Intervention 1: *n* = 21 Intervention 2: *n* = 20	Primary fibromyalgia	Mud bath therapy: 31–69 years range, mean ± SD 52.81 ± 10.26 Balneotherapy: 42–68 years range, mean ± SD 54 ± 7.22

As shown in Table 2, selected articles reported hydrotherapy (1 study), balneotherapy (3 studies), and mud therapy (1 study) interventions. Water temperature ranged from 36 to 42 °C, while mud was applied at 47 °C. Control interventions were the same water but at a lower temperature in the hydrotherapy study and non-mineral water at the same temperature in two balneotherapy studies. Intervention frequency ranged from 1 to 12 sessions, and duration ranged from 10 to 20 min. All balneotherapy/mud therapy studies were carried out at the Montecatini spa in Italy, therefore using the same waters.

**Table 2:** Characteristics of the interventions carried out in the studies included in the systematic review

Study	Intervention type	Characteristics of water and/or peloids	Intervention duration and frequency
Kurabayashi et al. 2001	Hydrotherapy, head-out hot water immersion	Intervention: tap water, 42 °C Control intervention: tap water, 37 °C	Intervention: one session, 10 min Control intervention: one session, 10 min
Marazziti et al. 2007	Balneotherapy	Montecatini spa (Italy) Intervention: alcalinic salso-sulphidric ozonized water, 36 °C Control intervention: non-mineral water, 36 °C	Intervention: one session, 20 min Control intervention: one session, 20 min
Baroni et al. 2012	Balneotherapy	Montecatini spa (Italy) Intervention: alcalinic salso-sulphidric ozonized water, 36 °C Control intervention: non-mineral water, 36 °C	Intervention: one session, 20 min Control intervention: one session, 20 min
Bazzichi et al. 2013	Balneotherapy and mud-bath therapy	Montecatini Terme Spa (Italy), salse-sulfate alkaline waters, formed of sodium chlorides and sodium and magnesium sulfates Mud-bath therapy: whole-body application of mud at 47 °C followed by bath in thermal water at 38 °C Balneotherapy: bath in thermal water at 38 °C	Mud-bath therapy: 12 sessions, once daily for 6 days/week, 20 min (10 min of mud therapy and 10 min of immersion in thermal water) Balneotherapy: 12 sessions, once daily for 6 days/week, 20 min

The effects of hydrotherapy, balneotherapy, or mud therapy interventions are summarized in Table 3. Three of the studies focused on determinations related to serotonin, and one study focused on dopamine analysis. Serum dopamine concentrations increased after hydrotherapy at different temperatures in healthy individuals (Kurabayashi et al. 2001). Serotonin studies were carried out in the same laboratory, using the same variables and measurement methods: determination of serotonin or 5-HT platelet transporter (SERT) maximal binding capacity (Bmax) and dissociation constant (Kd). Two of these studies (the ones carried out in healthy subjects) showed the same results. The affinity of SERT to its ligand increased after balneotherapy; thus, a lower amount of ligand seems to be required to obtain the protein saturation. The control group bathing in non-mineral water did not present any changes in SERT parameters (Marazziti et al. 2007; Baroni et al. 2012). However, in the study carried out on fibromyalgia patients, no significant changes in SERT were observed after balneotherapy or mud-bath therapy (Bazzichi et al. 2013).

**Table 3:** Effects of the interventions evaluated (hydrotherapy, balneotherapy, mud therapy) on the serotonin or dopamine parameters analyzed in the studies included in the systematic review.

Study	Variables	Measurement method	Comparison	Results
Kurabayashi et al. 2001	Serum dopamine concentration (pmol/L)	Radioimmunoassay	Before (0 min) vs. 15 and 30 min after the start of water immersion	Dopamine concentration increased after 15 min in intervention group (mean ± SD 71.2 ± 20 vs. 112.9 ± 23.7, *p* < 0.01) and control group (53.2 ± 24.7 vs. 96.5 ± 39, *p* < 0.05); but returned to pre-immersion values after 30 min in intervention group (71.2 ± 20 vs. 76.5 ± 25.3) and control group (53.2 ± 24.7 vs. 85.4 ± 32.2)
Marazziti et al. 2007	5-HT platelet transporter (SERT) maximal binding capacity (Bmax; fmol/mg protein) and dissociation constant (Kd; nM)	Analysis of specific binding of (3)H-paroxetine to platelet SERT. Equilibrium-saturation binding data, Bmax and Kd, were obtained by means of the Scatchard analysis	10 min before (t0) vs. 30 min after (t1) baths Intervention vs. control at t0 and t1	Intervention: Bmax did not change at t1 (mean ± SD 918 ± 234 vs. 909 ± 276); Kd values were lower at t1 (0.043 ± 0.012 vs. 0.034 ± 0.009, *p* = 0.006) Control: no changes in Bmax (1095 ± 194 vs. 1087 ± 1670) or Kd (0.058 ± 0.02 vs. 0.06 ± 0.009) Kd values at t1 were lower in the intervention group vs. control (*p* = 0.003)
Baroni et al. 2012	5-HT platelet transporter (SERT) maximal binding capacity (Bmax; fmol/mg protein) and dissociation constant (Kd; nM)	Analysis of specific binding of (3)H-paroxetine to platelet SERT. Equilibrium-saturation binding data, Bmax and Kd, were obtained by means of the Scatchard analysis	10 min before (t0) vs. 30 min after (t1) baths Intervention vs. control at t0 and t1	Intervention: Bmax did not change at t1 (mean ± SD 1105 ± 270 vs. 989 ± 246); Kd values were lower at t1 (0.052 ± 0.015 vs. 0.032 ± 0.008, *p* = 0.001) Control: no changes in Bmax (1295 ± 214 vs. 1184 ± 370) or Kd (0.056 ± 0.03 vs. 0.06 ± 0.008) Kd values at t1 were lower in the intervention group vs. control *(p* = 0.002)
Bazzichi et al. 2013	5-HT platelet transporter (SERT) maximal binding capacity (Bmax; fmol/mg protein) and dissociation constant (Kd; nM)	Analysis of specific binding of (3)H-paroxetine to platelet SERT	Baseline (t0) vs. 2 weeks after (t1) and 12 weeks after (t2) interventions	Balneotherapy: no changes in Bmax (mean ± SD 912 ± 334 vs. t1 1011 ± 466 or t2 913 ± 471) or Kd (0.035 ± 0.008 vs. t1 0.06 ± 0.07 or t2 0.08 ± 0.07) Mud-bath therapy: no changes in Bmax (1122 ± 443 vs. t1 1201 ± 371 or t2 1165 ± 473) or Kd (0.048 ± 0.044 vs. t1 0.05 ± 0.05 or t2 0.08 ± 0.06)

Overall, the methodological quality of the selected studies was poor. Seventy-five percent of the studies obtained the lowest score possible (zero), and only one study obtained 3 points out of 5, which is considered an acceptable quality (Table 4). However, we have to consider the particular characteristics of balneotherapy studies, because sometimes, it is very complicated or not feasible to carry out double-blind studies, for example when two different therapies are being compared (balneotherapy and mud therapy).

**Table 4:** Methodological quality of the studies as evaluated by the JADAD scale. 1a, was the study described as randomized?; 1b, was randomization appropriate?; 2a, was the study described as double-blind?; 2b, was blinding appropriate?; 3, was there a description of withdrawals and dropouts?

Study	1a	1b	2a	2b	3	Total score
Kurabayashi et al. 2001	0	-	0	-	0	0
Marazziti et al. 2007	0	-	0	-	0	0
Baroni et al. 2012	0	-	0	-	0	0
Bazzichi et al. 2013	1	1	0	-	1	3

## Discussion

There are two independent serotonin systems: brain and periphery. They remain separated due to (1) the tissue-specific expression of two different isoforms of the rate-limiting enzyme tryptophan hydroxylase (TPH) involved in the synthesis of 5-HT and to (2) the inability of serotonin to cross the blood-brain barrier. Almost 95% of serotonin is outside the central nervous system, as it is produced by enterochromaffin cells in the digestive tract and then taken up and stored mainly by platelets via the serotonin transporter SERT, but it is also stored by lymphocytes, monocytes, macrophages, and mast cells (Casas-Engel and Corbí 2014; Wu et al. 2019). Platelets are major regulators of circulating serotonin concentrations. Under physiological conditions, systemic concentration of serotonin is low, but upon platelet activation (for example in inflammatory conditions and after pro-inflammatory stimuli such as LPS or IFN-γ), serotonin is released from these cells, greatly increasing plasmatic concentration of this mediator, especially around sites of inflammation (Casas-Engel and Corbí 2014). Thus, serotonin is intricately linked to the regulation of inflammatory processes and immune responses in various physiological and pathological contexts. More specifically, serotonin modulates (stimulating or inhibiting) the effector functions of granulocytes, B and T lymphocytes, monocytes, dendritic cells, and tissue-resident macrophages (Casas-Engel and Corbí 2014). Several immune cell types are able to synthesize, transport, store, and respond to serotonin through serotonin receptors expressed in these cells, although the exact role of many of these receptors is still unclear (Wu et al. 2019) due to the tissue- and cell-specific pattern of expression as well as distinct signaling capacity of the different subtypes of serotonin receptors (Nieto et al. 2021). Therefore, the pleiotropic, numerous actions of serotonin on immune cells and the inflammatory response can be different depending on the serotonin receptor subtype, and cell maturation stage... among several other factors (Casas-Engel and Corbí 2014). Indeed, peripheral serotonin modulates leukocyte migration into sites of inflammation, and the elevated levels of serotonin found at inflamed sites contribute to regulating cytokine production during inflammation promotion and resolution (Nieto et al. 2021). In turn, activated immune cells can also influence serotonin synthesis and secretion in the gut (Spohn and Mawe 2017). In this context, altered peripheral serotonin levels have been shown to be involved in the development and resolution of immunity/inflammation-related pathologies like pulmonary arterial hypertension, atopic dermatitis, allergic asthma, systemic sclerosis, amyotrophic lateral sclerosis, and inflammatory gut disorders and also regulate cancer angiogenesis, neuroendocrine neoplasms proliferation, and arthritis (Nieto et al. 2020, 2021). In rheumatoid arthritis (RA), synovial fluid and circulating serotonin levels are elevated; in fact, it has been reported that intra-articular injection of serotonin in models of arthritis causes joint inflammation and pain, while its depletion attenuates disease severity (Shajib and Khan 2015). Tropisetron has also been reported to completely block serotonin-induced overexpression of prostaglandin E2, as well as to downregulate TNF-α and IL-1β (Seidel et al. 2008). The magnitude of arthritis has also been shown to be limited by the blockade of 5-HT2A receptors with ketanserin and ritanserin (Pertsch et al. 1993). Therefore, blockade of serotonin action seems to have anti-inflammatory effects in inflammatory rheumatic diseases. Recent studies have demonstrated a role for circulating serotonin as a regulator of bone mass (via osteoblastogenesis and osteoclastogenesis modulation), inhibiting bone formation (Chabbi-Achengli et al. 2016; Bernardes et al. 2017): when circulating concentrations of serotonin are decreased or increased, increases and decreases in bone volume have been observed (Spohn and Mawe 2017). In fact, RA patients treated with selective serotonin reuptake inhibitors (SSRIs) present increased hip and wrist fractures and decreased bone mineral density (Bernardes et al. 2017; Wadhwa et al. 2017). Furthermore, peripheral serotonin is involved in pain mechanisms, acting together with other proinflammatory mediators to contribute to injury and inflammation-induced pain and hyperalgesia (Haleem 2018). In this way, peripheral serotonin has been proposed as a new target for researchers and clinicians for the treatment of different immune/inflammatory diseases (Banskota and Khan 2022). Thus, spa therapy could be a potential strategy for the modulation of peripheral serotonin levels in several pathologies.

Regarding dopamine immunoregulation, immune cells express dopamine receptors and other dopamine-related proteins, allowing immune cells to actively respond to this molecule, for example modulating cytokine secretion, cell adhesion, cytotoxicity, chemotaxis, and phagocytosis. In turn, these immune responses can affect dopaminergic signaling both centrally and peripherally (Matt and Gaskill 2020), and also, dopamine produced and released by immune cells themselves can act as an autocrine/paracrine mediator on immune cells as well as on neighboring cells (Pinoli et al. 2017). Thus, dopaminergic immunoregulation is being increasingly considered an essential part of proper immune function (Pinoli et al. 2017; Matt and Gaskill 2020). However, the exact mechanisms and functions of dopaminergic signaling have yet to be fully understood, due to the variety of dopamine receptor subtypes with distinct intracellular signaling pathways leading to different effects on immune cells, consequently eliciting a wide range of inflammatory responses (Feng and Lu 2021). Dysfunction of the systemic or local dopaminergic system during inflammation has been found in various inflammatory diseases, such as Parkinson’s disease, inflammatory bowel disease, RA, systemic lupus erythematosus, and multiple sclerosis (Feng and Lu 2021). Recently, the importance of dopaminergic pathways has been highlighted in the pathophysiology of OA (Sheikhpour et al. 2020), and dopamine has even been proposed as a novel therapeutic agent for OA treatment (Lu et al. 2019). Therefore, spa therapy could potentially contribute to the immunomodulation of dopamine in rheumatic pathologies such as OA.

This systematic review aimed to evaluate the current scientific evidence about the role of spa therapy (including hydrotherapy, balneotherapy, and pelotherapy) on the neurotransmitters/hormones serotonin and dopamine. Four studies were included, and the results were heterogeneous due to the very different characteristics of each study. Results showed that, in young healthy individuals, serum dopamine concentrations acutely increased 15 min after one 10-min session of hydrotherapy, irrespective of water temperature (42 and 37 °C) but returned to basal levels after 30 min (Kurabayashi et al. 2001). Since no differences between bathing at different temperatures were found, authors suggested that acute dopamine changes were not derived from a thermal effect but from hydraulic pressure effects. On the other hand, the other three studies focused on serotonin, but used an indirect measurement: SERT Bmax and Kd. Two studies, using the same type of mineral-medicinal waters and carried out in young healthy individuals, showed that the affinity of SERT to its ligand acutely increased after one 20-min session of balneotherapy at 36 °C. This means that a lower amount of ligand seems to be required to obtain the protein saturation, but no clear and definitive conclusions about circulating or tissue concentrations of serotonin can be obtained from these results. The control groups bathing in non-mineral water at the same temperature did not show any changes in SERT parameters, which suggests that SERT acute changes could be directly related to the balneotherapy treatment and water composition, instead of being caused by water immersion itself and its physical effects (Marazziti et al. 2007; Baroni et al. 2012). The other study that focused on serotonin was carried out in an older group of fibromyalgia patients using the same type of mineral waters as the other two studies described above, and the same SERT parameters were evaluated. They were divided into two groups and each group underwent a different treatment: balneotherapy or mud-bath therapy. However, the temperatures used were 47 °C for the mud and 38 °C for the water, and patients received 12 20-min sessions instead of a single 20-min session. Moreover, no acute evaluation was performed, since samples for SERT analysis were obtained 2 and 12 weeks after the therapy. Results showed that no significant changes in SERT were observed after balneotherapy or mud-bath therapy at either time point (Bazzichi et al. 2013). Inconsistent SERT results could then be due to the heterogeneity of the study protocols: acute vs. long-term assessment, number of balneotherapy sessions, water and mud temperatures, pathology of the participants, etc. These findings highlight the usefulness of the evaluation of changes in serotonin and dopamine levels that could potentially be mediating the previously reported improvements in inflammatory responses (Cheleschi et al. 2022) and in mental health (Yang et al. 2018) after these therapies, which is particularly significant in light of the link between alterations in serotonin and dopamine concentrations, chronic low-grade inflammation, and various health conditions.

Taking all of the above into account, it is clear that a very low number of studies on the topic have been found. There was a very high heterogeneity of participants, intervention protocols, and measures, making it very difficult to extract clear conclusions. Secondly, the majority of the studies presented low methodological quality. Moreover, in our opinion, the serotonin parameters evaluated (binding to SERT) are not the most appropriate to reflect the potential physiological effects of these therapies. Direct measurements such as serum or plasma concentrations can more accurately reflect the physiological mechanisms of effectiveness underlying the clinical benefits of balneotherapy and its modalities, particularly considering the association between serotonin and inflammatory responses. While all those aspects could be considered limitations of our work, the selected studies reflect the current state of the literature and the scarcity of research in this particular area, indicating the need for more comprehensive and homogeneous studies with higher methodological quality on these biomarkers (along with other mediators related to the neuroendocrine response). In fact, the results obtained from article selection have been useful to reveal the gaps in knowledge concerning these neurotransmitters and hormones in the context of spa therapy, leading our group to carry out a pilot study evaluating the effect of balneotherapy (with the use of peloids) on the systemic concentration of peripheral serotonin and dopamine in OA patients.

## Conclusions

A very low number of studies assess the effects of spa therapy (hydrotherapy, balneotherapy, and mud therapy) on serotonin or dopamine function. These studies are very heterogeneous and present low methodological quality, making it difficult to draw clear conclusions. Further studies are needed to evaluate the potential effects of these therapies on serotonin or dopamine function.
